# Comparison of Staging Methods for Treatment-Resistant Depression: Chart Review

**DOI:** 10.1192/j.eurpsy.2024.540

**Published:** 2024-08-27

**Authors:** K. B. Avanoğlu, N. Oktar Erdoğan, E. Ağaoğlu, K. Başar

**Affiliations:** ^1^Psychiatry, Yalova State Hospital, Yalova; ^2^Psychiatry, Pamukkale University Faculty of Medicine, Denizli; ^3^Psychiatry, Bahçelievler Medipol Hospital, İstanbul; ^4^Psychiatry, Hacettepe University, Faculty of Medicine, Ankara, Türkiye

## Abstract

**Introduction:**

Treatment-resistant depression (TRD) lacks a universally consistent definition due to varied interpretations despite attempts to define it based on inadequate response or remission despite sufficient antidepressant treatment. There’s a crucial demand for a uniform definition and staging to streamline its effective management amid diverse treatment options and the complex nature of resistance. Five methods have emerged to define and classify treatment resistance reliably.

**Objectives:**

The aim of this study is to compare the five staging methods (Thase&Rush SM (T&R), European Staging Method (ESM), Maudsley Staging Method (MSM), Massachusetts General Hospital Staging Method (MHG-s), Conway Staging Method(Conway)) in assessing treatment resistance within a single sample.

**Methods:**

Retrospective analysis involved medical records of inpatient psychiatry clinic admissions at Hacettepe University between October 2012 and October 2014. Patients with a primary diagnosis of bipolar affective disorder, schizophrenia, other chronic psychotic disorders, dementia or cognitive disorders, alcohol and substance use disorders, and those with missing data were excluded.

**Results:**

Initial screening yielded a total of 115 patients. 64 patients were included in the study, 13 patients were excluded due to missing data, and 38 patients were excluded due to comorbidity.

**Table:** 

Characteristic	Total (N=64)	Last Episode Characteristics	Total (N=64)
Female - N(%)	44 (69)	Episode duration – month (mean ± SD)	13.75 ± 16.09
Age – yr (mean ± SD)	48.39 ± 18.81	Psychotic symptoms – N(%)	20 (31)
Married – N(%)	41 (64)	Anxiety symptoms – N(%)	24 (38)
Secondary school and over – N(%)	38 (59)	Suicidal attempt – N(%)	19 (30)
Employed – N(%)	16 (25)

**Table:** 

*TRD definition and staging method (N=55)*	T&R	ESM	MSM	MGH-S	Conway
Not resistant by this method	26 (47.3)	45 (81.8)	0 (0)	27 (49.1)	43 (78.2)
Identified by this method	29 (52.7)	10 (18.2)	55 (100)	28 (50.9)	12 (21.8)
Exclusively identified by this method	0 (0)	0 (0)	21 (38.2)	0 (0)	0 (0)
By this and one other method	27 (49.1)	0 (0)	11 (20)	5 (9.1)	0 (0)
By all methods	10 (18.2)	10 (18.2)	10 (18.2)	10 (18.2)	10 (18.2)
*Identified as TRD*					
Age of onset (mean ± SD)	40.28 ± 17.42	35.6 ± 18.27	40.44 ±18.38	40.07 ± 17.9	38.17 ± 17.71
ATHF score (mean ± SD)	7.55 ± 5.46	12.1 ± 6.51	4.93 ± 4.98	7.43 ± 5.69	11.08 ± 6.47
Last episode duration (month) (mean ± SD)	17.11 ± 17.25	22.10 ± 20.96	14.22 ± 17.08	16.33 ± 17.66	20.83 ± 19.25

**Conclusions:**

There is no universally agreed-upon definition for treatment resistance. In this sample, different definition and staging methods were employed to examine the similarities and differences in the clinical and treatment related characteristics of groups with TRD identified with each. The reasons and possible implication of concurrence and discordance between the methods will be discussed.

**Disclosure of Interest:**

None Declared

